# Study protocol of the RaPS study: novel risk adapted prevention strategies for people with a family history of colorectal cancer

**DOI:** 10.1186/s12885-018-4646-5

**Published:** 2018-07-06

**Authors:** Kaja Tikk, Korbinian Weigl, Michael Hoffmeister, Svitlana Igel, Matthias Schwab, Jochen Hampe, Stefanie J. Klug, Ulrich Mansmann, Frank Kolligs, Hermann Brenner

**Affiliations:** 1 Division of Clinical Epidemiology and Aging Research, German Cancer Research Center (DKFZ), Im Neuenheimer Feld 581, 69120 Heidelberg, Germany; 2 German Cancer Consortium (DKTK), German Cancer Research Center (DKFZ), Heidelberg, Germany; 3 Dr. Margarete Fischer-Bosch Institute of Clinical Pharmacology and University of Tübingen, Stuttgart, Germany; 4 University of Tübingen, Tübingen, Germany; 5 Department of Clinical Pharmacology, University Hospital, Tübingen, Germany; 6 Department of Biochemistry and Pharmacy, University of Tübingen, Tübingen, Germany; 7 Gastroenterology & Hepatology, Medical Klinic I, University Clinic Dresden, Technical University, Dresden, Germany; 8 Cancer Epidemiology, University Cancer Centre Dresden, Technical University Dresden, Dresden, Germany; 9 Epidemiology, Department of Sport and Health Sciences, Technical University of Munich, Munich, Germany; 10 Department of Medical Information Sciences, Biometry, and Epidemiology (IBE), Ludwig Maximilians Universität (LMU), Munich, Germany; 11 Department of Medicine II, University of Munich, Munich, Germany; 12 HELIOS Clinic Berlin-Buch, Berlin, Germany; 13 Division of Preventive Oncology, German Cancer Research Center (DKFZ) and National Center of Tumor Diseases (NCT), Heidelberg, Germany

**Keywords:** Colorectal cancer, Early detection, Screening, Family history

## Abstract

**Background:**

People aged 40–60 years with a family history (FH) of colorectal cancer (CRC) in 1st degree relatives (FDRs) have a 2- to 4-fold increased risk of CRC compared to the average risk population. Therefore, experts recommend starting CRC screening earlier for this high-risk group. However, information on prevalence of relevant colonoscopic findings in this group is sparse, and no risk adapted screening offers are implemented in the German health care system. For example, screening colonoscopy is uniformly offered from age 55 on, regardless of family history. Thus, we initiated a multicenter epidemiological study - the RaPS study (Risk adapted prevention strategies for colorectal cancer) – with the following aims: to determine the prevalence of having a FH of CRC in FDR in the German population aged 40–54 years; to investigate the prevalence of colorectal neoplasms among people with a FDR; and to develop risk-adapted prevention strategies for this high-risk group based on the collected information.

**Methods/Design:**

A random sample of 160.000 persons from the general population aged 40–54 years from the catchment areas of three study centers in Germany (Dresden, Munich and Stuttgart) are contacted to assess FH of CRC by an online-questionnaire. Those with a FH of CRC in FDRs are invited to the study centers for individual consultation regarding CRC prevention. Participants are asked to donate blood and stool samples and medical records of colonoscopies will be obtained. Prevalence of CRC and its precursors will be evaluated. Furthermore, genetic, epigenetic and proteomic biomarkers in blood and microbiomic biomarkers in stool will be investigated. Risk markers and their eligibility for risk adapted screening offers will be examined.

**Discussion:**

This study will provide data on the prevalence of colorectal neoplasms among persons with a FH of CRC in the age group 40–54 years, which will enable us to derive evidence based screening strategies for this high-risk group.

**Trial registration:**

This trial was registered retrospectively in the German Clinical Trials Register (DRKS) on 29th of December 2016: German Clinical Trials Register DRKS-ID: DRKS00007842.

## Background

Colorectal cancer (CRC) is the third most common cancer globally, and the fourth most common cancer cause of death [1]. There is increasing evidence that the burden of the disease can be limited to a large extent by screening [2], and screening programs are meanwhile implemented in an increasing number of countries [3, 4]. It is well known that people with a family history (FH) of CRC are at increased risk of the disease [5] and guidelines commonly recommend that first degree relatives of CRC patients should undergo earlier and more intensive screening [6]. However, data from large scale population-based studies on the prevalence of FH of CRC according to age, sex and other potential determinants, on the age and sex specific prevalence of colorectal neoplasms according to FH, on the use of CRC screening offers by those with a FH, and on the performance of potential noninvasive screening methods in this high risk group are sparse. In order to address these questions in detail, we initiated the RAPS study (Risk adapted prevention strategies for colorectal cancer) in the context of the German Cancer Consortium. Here, we describe the study design and summarize the study protocol.

### Objectives

This study aims to provide empirical evidence for the design and implementation of enhanced risk adapted CRC screening strategies and screening coverage for people with FH of CRC in Germany. In addition, the aim is to identify and evaluate novel biomarkers for risk stratification and early detection of CRC and its precursors among people with a FH of CRC.

In particular, the following primary research questions are addressed:

1. What is the prevalence of a history of CRC in 1st and 2nd degree relatives in the German population aged 40–54 years? To what extent are affected people aware of their increased risk and possibilities of early detection and prevention?
2. What are the prevalences of colorectal neoplasms (CRC, advanced adenomas and non-advanced adenomas), overall and by number, size, location, histopathological and molecular characteristics among people aged 40–54 years with a 1st degree relative (FDR) with CRC? How do these prevalences vary according to age, sex and other risk factors, and according to number, sex, and age at diagnosis of affected 1st and 2nd degree relatives?
3. To what extent can genetic, epigenetic, proteomic and microbiomic biomarkers predict the presence of colorectal neoplasms among people with a FH of CRC? To what extent can this information be used for effective risk stratification and personalized, targeted screening?
4. What are the implications for the need, design, effectiveness and cost-effectiveness of special screening offers for people with a positive family history of CRC? In particular, the implications of the findings for effective risk adapted, personalized secondary prevention of CRC are to be worked out.

## Methods/Design

**Fig. 1:**
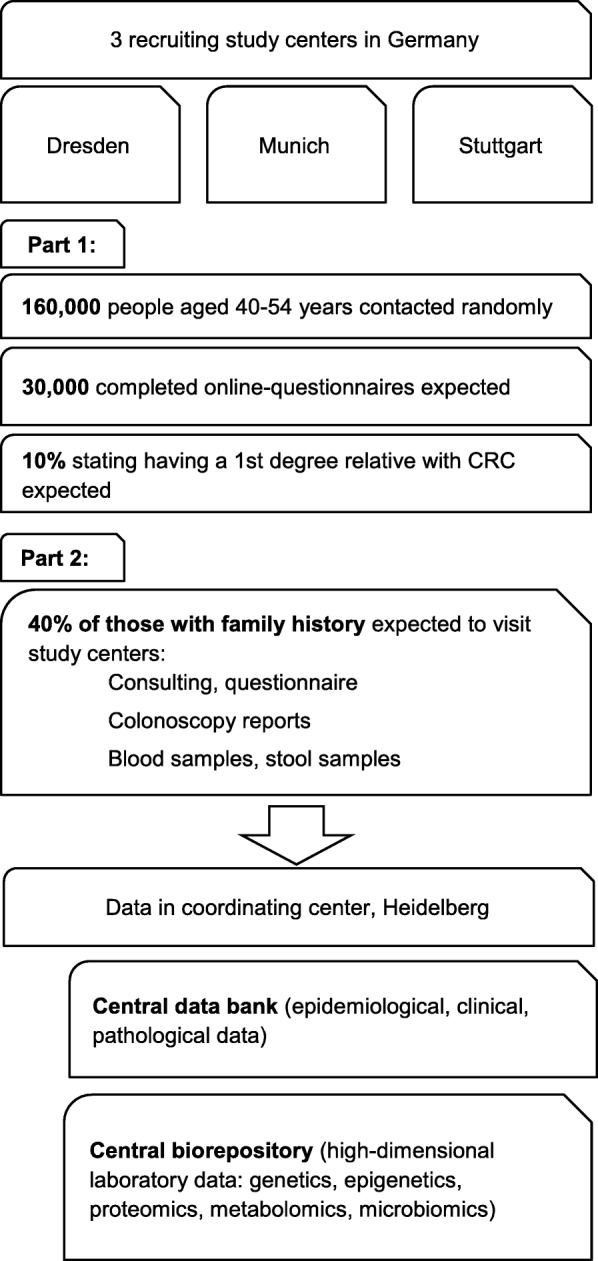
Study Flow Chart

The RAPS study is a multicenter cross-sectional study of the general population in Germany. The recruitment of the participants takes place in three large cities in Germany (Dresden, Munich, Stuttgart) and the study is conducted in two parts consisting of (i) an online-survey and (ii) in-depth personal consultation and examination (Fig. 1, study flow chart).

### Part 1 - Online survey

For the first study part, random samples (*n* = 40,000-60,000) of the general population aged 40–54 from the catchment areas of each of the 3 recruiting centers (Dresden, Munich, Stuttgart) are drawn from local population registration offices. The selected population is screened for the presence of increased CRC risk due to FH by an online questionnaire. From June 2015 onwards, invitation letters with personalized six-digit passwords (consisting of letters and numbers) which allow access to the online questionnaire are sent to 4000 to 6000 persons per recruitment city on a monthly basis, with a total of 160,000 persons being invited to complete the online questionnaire. The invitation letters include a scannable QR code which directly leads to the online questionnaire. Prior to the access to the questionnaire, participants have to confirm that they have read the participants’ information including information about data protection, which are provided online. After this, an online-informed consent is obtained. Persons who are not able or not willing to complete the online questionnaire are offered a hard copy of the questionnaire as well as printed copies of all necessary documents and a reply-paid envelope. In this case, after written informed consent and the ascertainment of completion of the questionnaire, the data is transferred to the database by study data managers in the coordinating center in Heidelberg. People who do not respond to the first invitation for three weeks are contacted two more times with reminders, each sent on average three weeks after the previous invitation.

After completion of the online questionnaire, all participants instantly receive personalized feedback regarding their individual risk factors for CRC. Risk factors reported by the participants are automatically processed and coded after submission of the online questionnaire, and a computer based algorithm is generated based on the information about these risk factors. This automatically received feedback, prepared in collaboration with the German Cancer Information Service, provides information and recommendations regarding both common modifiable risk factors for CRC prevention, including smoking, alcohol consumption, body mass index, red meat intake, physical activity, as well as non-modifiable risk factors such as age, syndromes and diseases (e.g. Lynch syndrome, familial adenomatous polyposis, and inflammatory bowel diseases). Persons who complete hard-copies of the questionnaire receive the feedback with regular mail.

### Part 2 - In-depth personal consultation and examination in study centers

In addition to the feedback to their individual risk factors for CRC prevention, participants reporting a FH of CRC in a FDR automatically receive information on the second part of the study. These participants are contacted again either by letter, e-mail or by phone (if they provided a valid e-mail-address or telephone number in their online-questionnaire), and are offered to take part in an individual consultation regarding CRC prevention and examination regarding CRC risk in the study centers in their catchment areas. During the consultation, performed by trained gastroenterologists, persons are invited to participate in the second part of the study which includes an additional comprehensive questionnaire, the donation of blood samples, stool samples and a qualitative fecal immunochemical test (FIT). In addition, if recommended by gastroenterologists, participants are offered a screening colonoscopy either in the study centers or by cooperating private gastroenterologists in the study region.

### Inclusion and exclusion criteria

Inclusion criteria for the first study part comprise (a) age between 40 and 54 years, (b) living in the catchment areas of the three study centers and being included in the random samples drawn from the registration office, and (c) having sufficient German skills to understand the participants’ information and to complete the online questionnaire.

Inclusion criterion for the second study part is having a self-reported FH of CRC in at least one FDR as stated in the online questionnaire. Persons who were diagnosed with CRC prior to the study are excluded for the second part of the study.

## Data collection

### Questionnaire data

The online questionnaire in the first study part consists of questions about previously diagnosed diseases (e.g. cardiovascular diseases, diabetes, cancer), previously conducted prevention examinations (e.g. general health examination, skin cancer screening, mammography / prostate-specific antigen testing), previously conducted screening examinations for the colon (FIT, lower gastrointestinal exam, colonoscopy), FH of CRC in FDR (i.e. mother, father, sibling; if FH in FDR was present, age of diagnosis was obtained), FH of CRC in second-degree relative (SDR), lifestyle factors (e.g. smoking, alcohol consumption, nutrition, physical activity, intake of non-steroidal anti-inflammatory drugs/aspirin, intake of hormone replacement therapy for women) and personal information (such as sex, age, height, weight, education, household size). If participants state having had previous colonoscopies, permission to request colonoscopy and histological reports from the treating physician is asked for.

Participants of the second study part are asked to complete an additional questionnaire with a more comprehensive ascertainment of the individuals’ family history (e.g. age at diagnosis for relatives, how many relatives are affected, total family size).

### Blood and stool samples

After individual consultation and signed informed consent for the second part of the study, blood samples (2 × 9 ml in serum vacutainer, 2 × 9 ml in EDTA vacutainer, 36 ml in total) are drawn immediately at the study centers. All blood samples are processed within two hours after donation according to a standardized operation procedure. After blood sampling, both serum vacutainers and one EDTA vacutainer are centrifuged for ten minutes with 2500 g at 4 °C, before they are stored at − 80 °C in 0.5 and 1 ml aliquots. The second EDTA vacutainer is centrifuged for 20 min with 1300 g at 4 °C. The supernate is further centrifuged for ten minutes with 15,000 g at 10 °C. The resulting supernate is stored at − 80 °C in 1 ml aliquots.

The participants receive a prepared stool sampling kit, which consists of two stool collection tubes including RNA later and one FIT (ImmoCARE-C, cut-off 50 ng/ml, Care diagnostic©). The FIT used in this study is a commercially available and validated test. The participants are asked to collect all stool samples from the same stool (no dietary restrictions, approximately 1 g each) and send it via regular mail to the coordinating center in Heidelberg with a prepaid envelope. If a participant decides to undergo colonoscopy, stool samples are taken before bowel preparation. The FIT is analyzed in the laboratory of the coordinating center in Heidelberg, and participants are informed about the test result in case of a positive FIT.

### Data collection and documentation in the coordinating center

All collected data and blood samples are gradually transferred from study centers to a central databank in the coordinating center in Heidelberg. The information collected from colonoscopy and histology reports is entered into a standardized study database by trained staff in the coordinating center, using double data entry by two independent staff members. Data entries are checked for inconsistencies through comparison of the corresponding data sets. In case of differences in data sets, original reports are checked for validation.

Documentation of information collected in the questionnaire (part 2) include automated scanning of the questionnaires, optical verification of the scans by trained staff, and comprehensive plausibility checks prior to statistical analysis. All collected information is stored at the coordinating center.

The blood and stool samples are stored at − 80 °C in a central biorepository at the coordinating center until further analyses.

### Sample size calculation

Expected sample size for study part 1 includes 3 × 10,000 = 30,000 online-questionnaire responses, which would enable the most comprehensive analyses of the occurrence of patterns of familial CRC risk on the population level available to date. These analyses will enable the evaluation of risk adapted screening strategies even for small, specific risk groups with the necessary precision.

Expected sample size for research questions 2 and 3 will include around 3 × 400 = 1200 women and men aged 40–54 with CRC in a FDR for whom extensive questionnaires, colonoscopy and histopathology reports, as well as biospecimens (blood, stool) will be available. With an expected prevalence of advanced neoplasms detected and removed at colonoscopy of 10% (*n* = 120), approximately half of which would be expected to develop into clinically manifest CRC within 10 years in the absence of detection and removal, this study will prevent a mentionable number of CRCs occurring at relatively young age. The sample size will furthermore ensure the estimation of the prevalence of colorectal neoplasms, as well as calculation of performance indicators of risk discrimination and risk stratification (e.g. area under the curve, numbers needed to screen) with adequate precision, even for relevant subgroups defined by age, sex, and other risk factors.

In the light of the fact that the current study is the first large scale epidemiological study trying to use this two-stepped approach for recruiting the participants with FH of CRC and that the current evidence regarding associated response rates is very limited, our above described expected sample size is calculated based on educated guess. To ensure the targeted sample size, several efforts to drive the response rates towards our expectation are considered and actively followed (e.g. increase of invited study population or reminders to participate).

### Statistical analysis

Prevalence of CRC and its precursors (in particular advanced adenomas and non-advanced adenomas as well as serrated polyps, overall and according to number, size, location, and histopathological characteristics) will be evaluated according to age and sex of participants, as well as number, type and age at diagnosis of affected 1st and 2nd degree relatives and other risk factors. Based on these results, implications for screening strategies (including cost-effectiveness) will be evaluated. In particular, the expected number of prevented CRCs and CRC deaths, and the expected cost savings by early detection and prevention of CRCs will be computed and related to the costs of screening at ages 40–54 in this high risk group. Recommendations will be worked out for risk adapted CRC screening offers for the high risk population of people with a positive FH of CRC.

## Discussion

We initiated a large-scale, two-phased, multicenter population-based epidemiological study within the German Cancer Consortium in order to provide solid empirical evidence for risk adapted, enhanced CRC screening strategies for persons with a FH of CRC. This study provides, for the first time in Germany, the necessary data for deriving evidence based screening guidelines for the high risk group of people with a FH of CRC. Active engagement of the investigators in guideline and policy development on the national and international level will ensure translation of results into practice.

Furthermore, due to detection and removal of advanced neoplasms at colonoscopy of participants in this study, it is expected to prevent a number of cases of CRC at relatively young age. If translated in risk adapted screening offers on the national level, a substantial proportion of the approximately 15,000 CRCs occurring before age 65 in Germany annually and their treatment costs could be prevented. The comprehensive risk factor, genetic, epigenetic, proteomic and microbiomic profiling will provide the basis for effective risk stratification and risk adapted screening strategies in this high risk population.

In order to further enhance the empirical basis for risk adapted, personalized prevention in this high risk group, the potential for enhanced risk stratification based on novel genetic and proteomic biomarkers from blood samples and microbiomic biomarkers from stool sample will be evaluated. For genetic profiling, Infinium® Global Screening Array by Illumina, which covers > 700,000 single nucleotide polymorphisms (SNPs) will be used. Imputation based on whole genome sequencing data will yield several millions SNPs which can be analyzed accordingly [7]. For high throughput high dimensional proteomic analysis, the newly launched protein biomarker panels from OLINK Bioscience (Sweden) will be used, allowing to analyze a large number of proteins related to different biological mechanisms, such as angiogenesis, cell-cell signaling, growth control and inflammation. For microbiome analysis, DNA extraction with 16S rRNA gene sequencing and shotgun metagenomics sequencing at the European Molecular Biology Laboratory (EMBL) GeneCore facility, using state-of-the-art sequencing techniques with a special workflow for microbiomics, will be applied.

## Abbreviations

CRC: Colorectal cancer
DNA: Desoxyribonucleic acid
EDTA: Ethylenediaminetetraacetic acid
EMBL: European Molecular Biology Laboratory
FDR: First-degree relative
FH: Family history
FIT: Fecal immunochemical test
QR code: Quick response code
rRNA: Ribosomal ribonucleic acid
SDR: Second-degree relative
SNP: Single nucleotide polymorphism

## References

[1] GLOBOCAN 2012 v1.0, Cancer Incidence and Mortatlity Worldwide: IARC CancerBase No. 11 [Internet]. [http://globocan.iarc.fr]. Accessed 12 Dec 2013.

[2] Brenner H, Stock C, Hoffmeister M (2014). Effect of screening sigmoidoscopy and screening colonoscopy on colorectal cancer incidence and mortality: systematic review and meta-analysis of randomised controlled trials and observational studies. BMJ.

[3] Stock C, Ihle P, Schubert I, Brenner H (2011). Colonoscopy and fecal occult blood test use in Germany: results from a large insurance-based cohort. Endoscopy.

[4] Shapiro JA, Klabunde CN, Thompson TD, Nadel MR, Seeff LC, White A (2012). Patterns of colorectal cancer test use, including CT colonography, in the 2010 National Health Interview Survey. Cancer Epidemiol Biomark Prev.

[5] Lowery JT, Ahnen DJ, Schroy PC, Hampel H, Baxter N, Boland CR, Burt RW, Butterly L, Doerr M, Doroshenk M (2016). Understanding the contribution of family history to colorectal cancer risk and its clinical implications: a state-of-the-science review. Cancer.

[6] Short MW, Layton MC, Teer BN, Domagalski JE (2015). Colorectal cancer screening and surveillance. Am Fam Physician.

[7] Li Y, Willer C, Sanna S, Abecasis G (2009). Genotype imputation. Annu Rev Genomics Hum Genet.

